# Supplementary material for: Living fabrication of functional semi-interpenetrating polymeric materials
Source: Nat Commun. 2021 Jun 8;12:3422. doi: 10.1038/s41467-021-23812-7 (PMC8187375; doi:10.1038/s41467-021-23812-7)
Supplement: Supplementary file 1 — Supplementary Information [file 41467_2021_23812_MOESM1_ESM.pdf]

Living fabrication of functional semi-interpenetrating polymeric materials

Zhuojun Dai[1,2+], Xiaoyu Yang[3], Feilun Wu[2], Lihua Wang[2], Kun Xiang[2], Pengcheng Li[1], Qingqing Lv[1], Jinhui Tang[1,4], Anders Dohlman[2], Lei Dai[1], Xiling Shen[2,5], Lingchong You[2,5,6+]

[1]Shenzhen Key Laboratory of Synthetic Genomics, Guangdong Provincial Key Laboratory of Synthetic Genomics, CAS Key Laboratory of Quantitative Engineering Biology, Shenzhen Institute of Synthetic Biology, Shenzhen Institutes of Advanced Technology, Chinese Academy of Sciences, Shenzhen 518055, China
[2]Department of Biomedical Engineering, Duke University, Durham, NC, 27708
[3]Systems, Synthetic, and Physical Biology Program, Rice University, Houston, TX, USA
[4]The Brain Cognition and Brain Disease Institute (BCBDI), Shenzhen Institutes of Advanced Technology, Chinese Academy of Sciences, Shenzhen, Guangdong, 518055, China
[5]Center for Genomic and Computational Biology, Duke University, Durham, NC, 27708
[6]Department of Molecular Genetics and Microbiology, Duke University School of Medicine, Durham, NC 27710
[+]Corresponding authors.
Shenzhen Institute of Synthetic Biology, Shenzhen Institutes of Advanced Technology, Chinese Academy of Sciences, Shenzhen 518055, China
E-mail: zj.dai@siat.ac.cn
Department of Biomedical Engineering, Duke University, CIEMAS 2355, 101 Science Drive, Box 3382, Durham, NC 27708, USA.
Tel.: +1 (919)660-8408; Fax: +1 (919)668-0795; E-mail: you@duke.edu

Supplementary Materials
Strains, circuits and media

Supplementary Figures, Tables and Videos
Supplementary Figures 1 to 17
Supplementary Tables 1 to 2
Captions for Supplementary Movies 1 to 2

Other Supplementary Materials for this manuscript include the following:
Supplementary Movies 1 to 2
Source data file

Supplementary Materials
Strains, circuits and media
Bacterial strains
Escherichia coli strain MC4100Z1(MC) and Nissle 1917(NI) were used for carrying the ePop circuit and a plasmid expressing protein monomers.
Circuit and plasmids

- ePop (ColE1 origin) was published previously[1]. Briefly, it was constructed using the *luxbox* region (140 bp upstream of *luxI*in *V. fischeri*) from p*lux*GFPuv and *E* gene coding sequence from φX174 (NEB). Each region was PCR-amplified and then joined together in an overlap PCR reaction. The '*lux* box-*E* gene' fragment was inserted into the AatII site of host vector pLuxRI2.

- His-T_3 (His-SpyTag-ELPs-SpyTag-ELPs-SpyTag), His-C_3 (His-SpyCatcher-ELPs-SpyCatcher-ELPs-SpyCatcher), His-T_2-mCherry (His-SpyTag-ELPs-mCherry-ELPs-SpyTag) and His-C_2 (His-SpyCatcher-ELPs-SpyCatcher), were constructed using the plasmids from the David A. Tirrell lab[2]. The fragments were cloned into the vector with T5 promoter and p15A origin.

- T_3-Bla was constructed using the synthesized fragment (His-SpyTag-ELPs-SpyTag-ELPs-SpyTag-ELPs-Bla) from Genewiz. The fragment was cloned into the vector with T5 promoter and p15A origin.

- The luminescence reporter plasmid was purchased from Addgene[3].

Growth media

The LB medium: 25 g LB Broth Powder (MO BIO Laboratories, Inc) was added into 1 L deionized H_2O. After autoclaving for 45 mins, the LB medium was stored at room temperature. The medium was supplemented with appropriate antibiotics (100 μg/mL chloramphenicol, 50 μg/mL kanamycin) when applicable.

The M9 medium: 1x M9 salts (48 mM Na_2HPO_4, 22 mM KH_2PO_4, 862 mM NaCl, 19 mM NH_4Cl), 0.4% glucose, 0.2% casamino acids (Teknova), 0.5% thiamine (Sigma-Aldrich), 2 mM $MgSO_4$, 0.1 mM $CaCl_2$ were added into 1 L deionized H_2O. M9 medium was adjusted with to pH = 7 by VWR Symphony SB70P pH Meter and filtrated through 0.22 μm filter.

Supplementary Figures, Tables and Videos

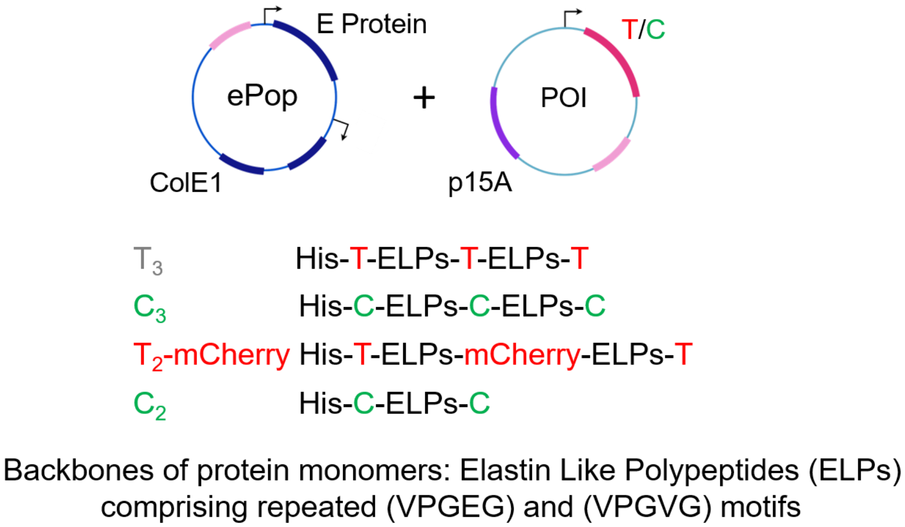

Supplementary Figure 1. Constructs to assemble the polymerized protein component. T and C indicate SpyTag and SpyCatcher, respectively. Cells were co-transformed with the ePop circuit to program the autonomous lysis, and the protein expression circuit to express the monomers.

Chitosan capsules

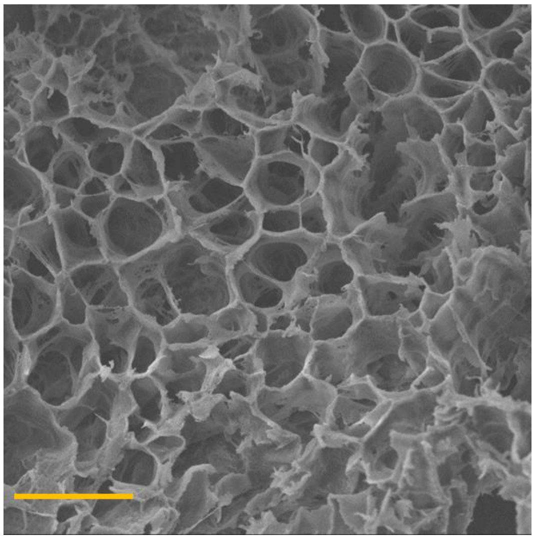

Supplementary Figure 2. SEM images show a crosslinked inner structure in chitosan capsules. To study the inner structure of the chitosan capsules, we dissected the capsules and examined the internal crosslinking structure by scanning electron microscope (SEM). The scale bar is 10 μm. The experiment was repeated more than three times independently with the similar results.

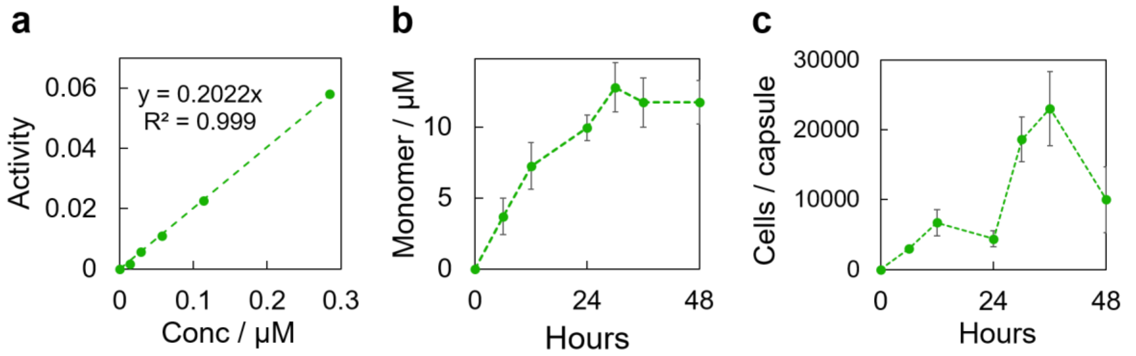

Supplementary Figure 3. Quantification of monomer concentration and bacterial viability inside capsules.

a. **Calibration using purified T_3-Bla.** T_3-Bla was expressed in *E. coli* and purified. The Bla activity of the T_3-Bla (different concentrations) was plotted against the concentration.

b. **Monomer concentration inside the capsules as the function of time.** The Bla activity of the released T_3-Bla from capsules was measured and then back calculated to the concentration in capsules at the function of time according to the calibration curve shown in panel A. The data showed that monomers concentration increased gradually with the time in the capsules and attained 7 μM and 10 μM in 12 and 24 hours. Experiments were done in triplicate and data represented mean values ± s.d, with error bars = Standard Deviation (n=3).

c. **The number of viable encapsulated bacteria as the function of time.** At different time points, the encapsulated bacteria were released and plated to determine the viable count. These data show that the cell number increased from ~10 cells to 10^4 cells per capsule in 24 hours. That is, in our system, bacterial growth and circuit function were not negatively affected by chitosan. Experiments were done in triplicate and data represented mean values ± s.d, with error bars = Standard Deviation (n=3).

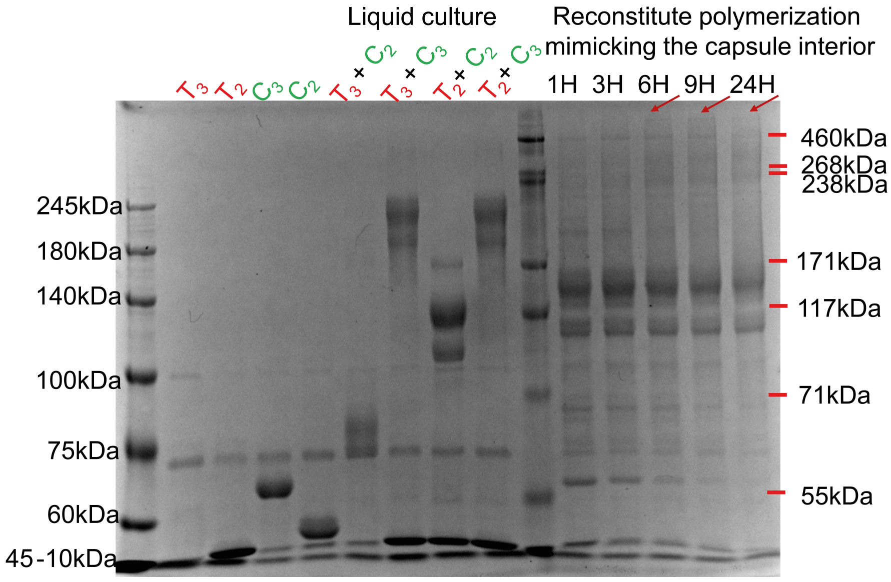

Supplementary Figure 4. Polymerization degree of the protein monomers at the concentration mimicking the capsules interior. Supplementary Figure 3 shows that the total concentration of T_3-Bla would accumulate to an equivalent of ~7 μM in 12 hrs, estimated from the total Bla activity. However, it is difficult to estimate the degree of polymerization directly inside the capsules. To overcome this limitation, we examined the polymerization between monomers at similar effective concentrations. In particular, we generated a mixture containing T_2-mCherry and C_3 (1:1 molar ratio, 7 μM in total), in a total reaction volume of 15 μL. The reaction was quenched at different time points (indicated by the top row) at -20 °C and assayed by SDS-PAGE (6% in the separating gel). Our results show that formation of polymerized products with molecular weights larger than 460kDa (pointed by the arrow) at this concentration, which is higher than the reaction product from the liquid culture (reaction in the supernatant which is harvested at 24 hrs). The experiment was repeated more than three times independently with the similar results.

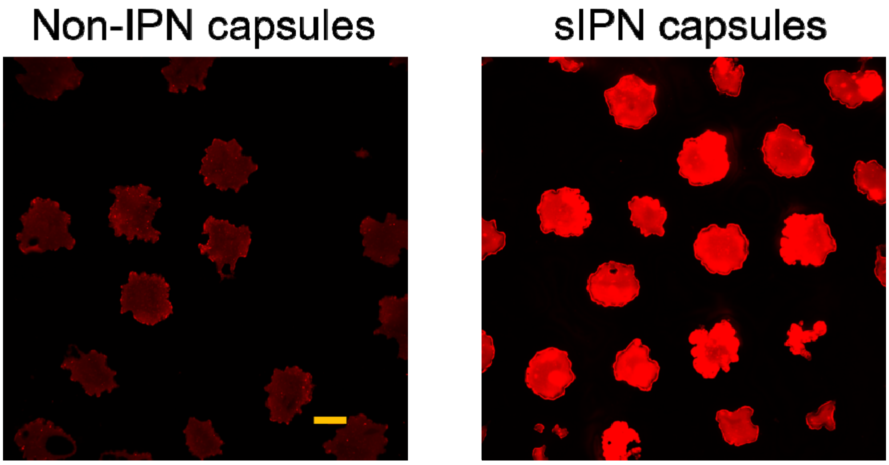

Supplementary Figure 5. Frozen section images indicate the immobilization of mCherry in living sIPN capsules. We first dehydrated the capsules, made frozen section (10 μm), and imaged the mCherry signal of living sIPN (carrying MC (T_2-mCherry) + MC (C_3)) and non-IPN (carrying MC (T_2-mCherry) + MC (T_3)) capsules using confocal microscopy. Compared with the non-IPN capsules (MC(T_2-mCherry) + MC (T_3)), the frozen sections of living sIPN capsules (carrying MC(T_2-mCherry) + MC (C_3)) show overall stronger mCherry signal, which is consistent with what we observed in videos taken by fluorescence microscopy (**Figure 2b**, **Supplementary Movies 1** and **2**). The scale bar is 100 μm. The experiment was repeated more than three times independently with the similar results.

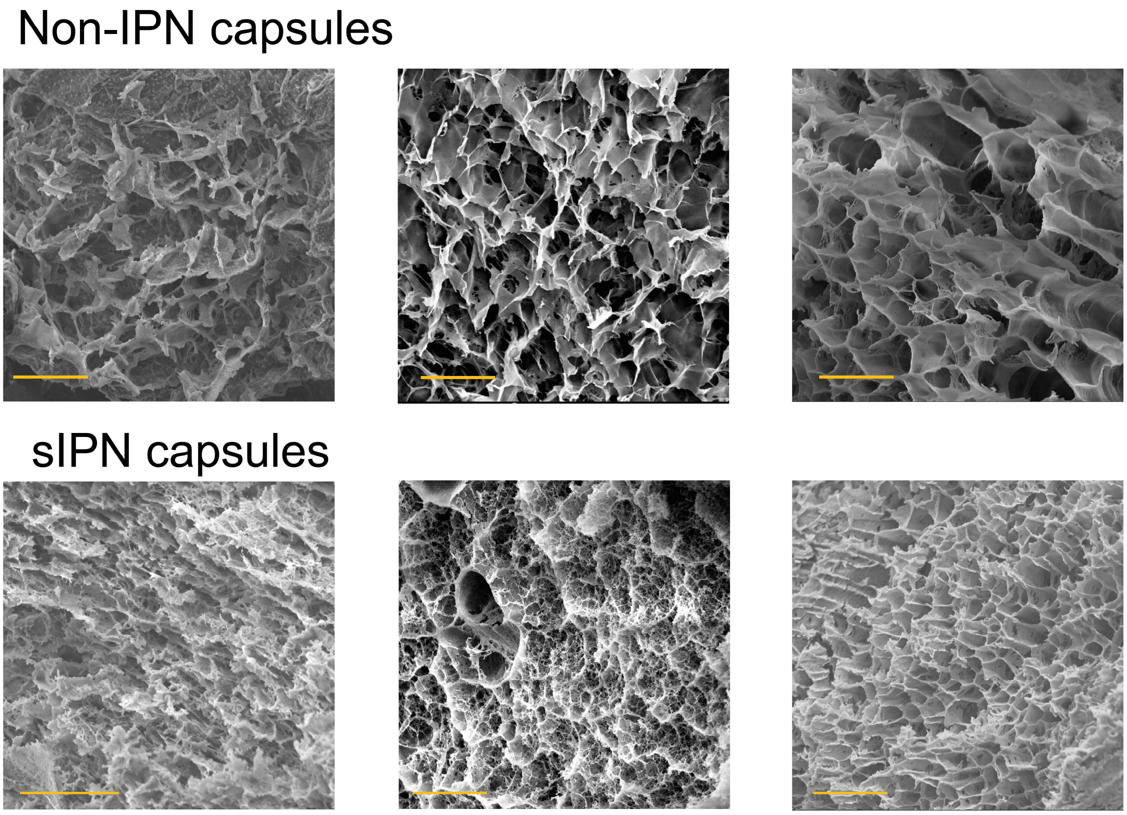

Supplementary Figure 6. SEM images show internal structure in living sIPN capsules has smaller pore size than non-IPN capsules. To study the inner structure of the IPN capsules, we dissected the capsules and examined the internal crosslinking structure by scanning electron microscope (SEM)[4]. We first lyophilized living sIPN and non-IPN capsules and examined these samples under SEM. Compared with non-IPN capsules (MC (T_2-mCherry) + MC (T_3)), the inner structure of the living sIPN capsules (carrying MC (T_2-mCherry) + MC (C_3)) has smaller pore size. The scale bar is 10 μm. The experiment was repeated more than three times independently with the similar results.

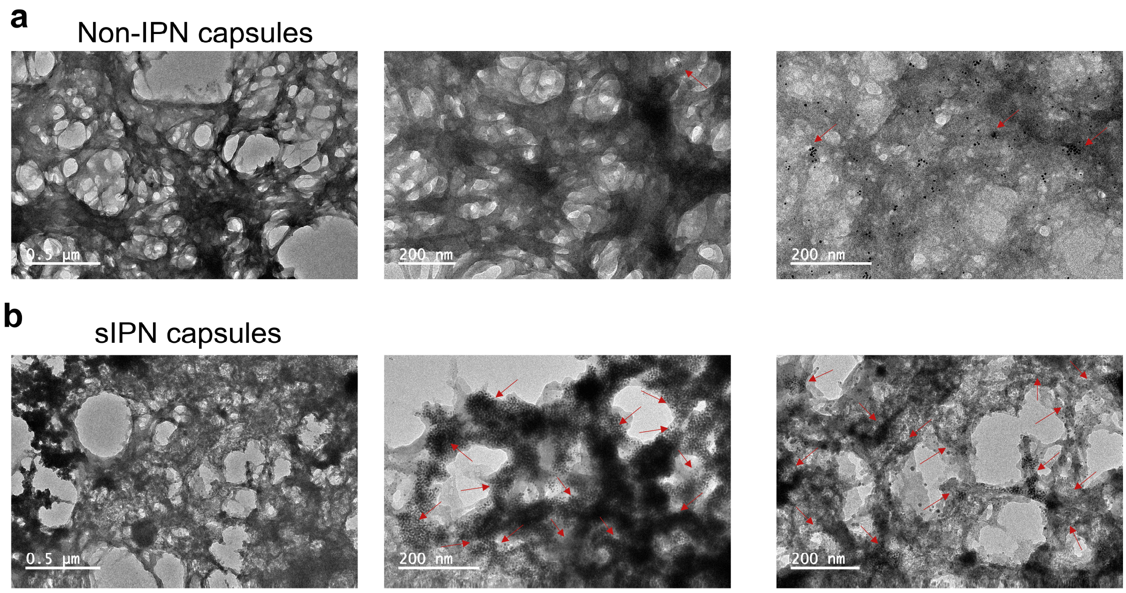

Supplementary Figure 7. TEM images confirmed the polymerized protein by differential labeling of gold nanoparticles. The protein monomers have a metal-binding tag (His-tagged), which can conjugate with Ni-NTA labeled gold nanoparticles (5 nm). Living sIPN (carrying MC (T_2-mCherry) + MC (C_3)) and non-IPN (carrying MC (T_2-mCherry) + MC (T_3)) capsules were first dehydrated, embedded and sliced into thin sections. The sections were placed on a coated copper grid and stained with Ni-NTA-AuNPs (5 nm) for TEM examination. Our results show the polymerized protein structure in living sIPN capsules as indicated by the densely alignment of the gold nanoparticles (pointed by arrows in **b**). In comparison, there is only sparse and scattered distribution of gold nanoparticles in the non-IPN capsules (pointed by arrows in **a**). The experiment was repeated more than three times independently with the similar results.

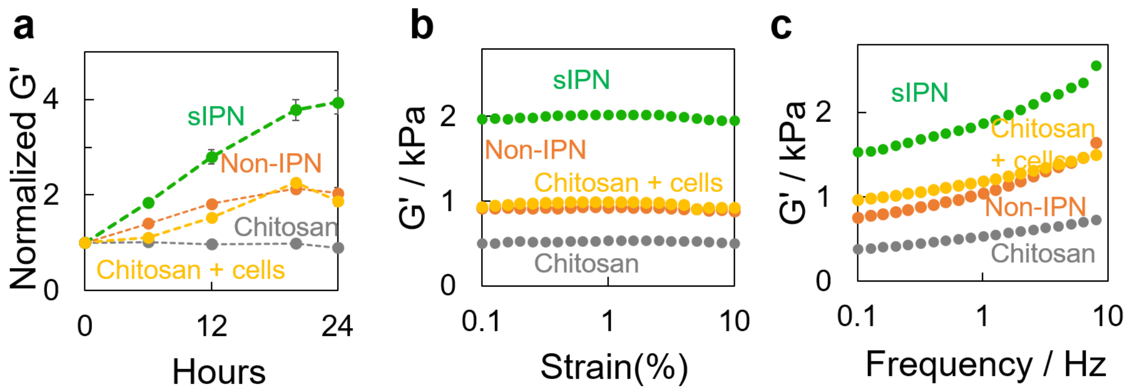

Supplementary Figure 8. sIPN reinforced the mechanical strength of the resultant material.

a. **The storage modulus of living sIPN capsules increased with time.** Living sIPN capsules were fabricated by encapsulating MC (T_2-mcherry) and MC (C_3) in chitosan. In the control group, MC (C_3) was replaced with MC (T_3). Living sIPN, non-IPN capsules, capsules containing cells but not producing any monomer (MC4100Z1(ePop)) and chitosan capsules (no cells) (same in below) were harvested at different time points and sent for storage modulus measurement using the strain sweep. The frequency was kept at 1.6 Hz (10 rad/sec), while the strain varied from 10% to 0.1%. The data was calculated based on the average of the storage modulus at different strains and normalized by the value at time zero. The storage modulus of the capsules containing no cells stayed constant after 24 hrs. That of the capsules containing cells (producing monomers) but no IPN formation and capsules containing cells (not producing monomers) increased by ~2 fold during the same time window, likely due to cell growth. In contrast, the storage modulus of living sIPN capsules increased by ~4 fold, suggesting an additional role in the formation of a semi-IPN. Storage modulus at each time point were calculated by averaging the values at 10 different strains between 0.1% to 10% and data represented mean values ± s.d, with error bars = Standard Deviation (n=10).

b. **Living sIPN capsules have a stronger storage modulus compared with other capsules at the constant frequency.** All materials were harvested at 24 hours. In the strain sweep, the frequency was kept at 1.6 Hz (10 rad/sec), while the strain varied from 10% to 0.1%.

c. **Living sIPN capsules have a stronger storage modulus compared with other capsules at the constant strain.** All materials were harvested at 24 hours. In the frequency sweep, the strain was kept as 5%, while the frequency varied from 0.1 to 10 Hz.

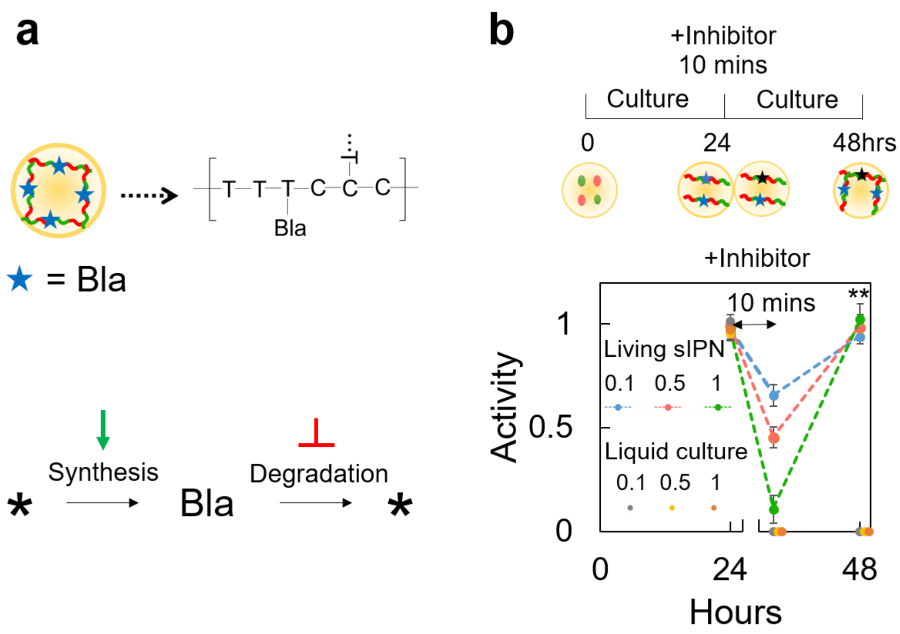

Supplementary Figure 9. Immobilization of functional proteins in living sIPN capsules enhances the functionality.

a. **Living sIPN capsules have an enhanced Bla activity.** Bla is immobilized inside the capsules due to the formation of sIPN. Bla activity is enhanced due to 1) the immobilization increases the stability of the enzyme, and 2) living cells inside capsules constitutively produce new Bla.

b. **Living sIPN capsules recovered the Bla activity after transient perturbations** *(Top: schematic; bottom: experimental data)*. MC(T_3-Bla) and MC(C_3) bacteria were mixed and inoculated in M9 (liquid culture), or encapsulated with chitosan (living sIPN capsules) and cultured in the same amount of M9 for 24 hours before the Bla enzymatic assay. The supernatant of the liquid culture (containing T_3-Bla), or the living sIPN capsules were incubated with clavulanic acid (0.1, 0.5 or 1 μg/mL) for 10 mins before the Bla enzymatic assay. For liquid culture, the Bla supernatant was first purified using His-tagged beads to remove the inhibitor, re-inoculated into the same amount of M9 and cultured for another 24 hours before the enzymatic assay. For living sIPN capsules, the treated capsules were collected and re-inoculated into the same amount of M9 nutrients and cultured for another 24 hours before the enzymatic assay. The data showed the Bla activity and was normalized by the control (supernatant or living sIPN capsules not treated by clavulanic acid). When clavulanic acid was supplemented for 10 mins, living Bla sIPN capsules could partially stabilize the Bla function. All the clavulanic acid treated living Bla sIPN capsules resumed the activity after another 24 hours culture; while the supernatant (containing Bla before treatment) did not regain any enzymatic activity. Experiments were done in triplicate and data represented mean values ± s.d, with error bars = Standard Deviation (n=3). Significance is defined as $^{**}p < 0.01$ ($p = 0.0004$, 0.001 and 0.002 for 0.1, 0.5 or 1 μg/mL clavulanic acid concentration) by the two-sided Student's two-sample t-test assuming unequal variances, compared with the value for the liquid culture. The x-axis is not to scale.

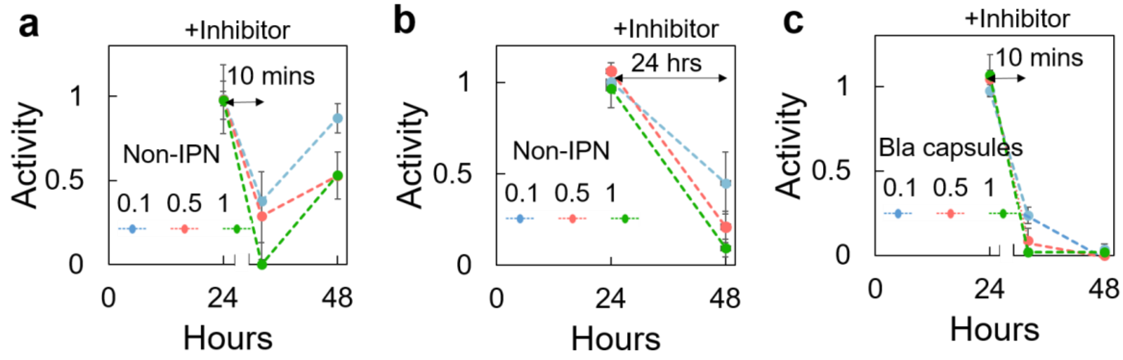

Supplementary Figure 10. Living non-IPN capsules producing Bla or capsules containing pure Bla but no bacteria had partial or no recovering capacity in response to transient inhibition.

a. **Living non-IPN capsules producing Bla partially recovered Bla activity after transient perturbation.** When clavulanic acid was supplemented for 10 mins (transient perturbation), living non-IPN capsules producing Bla remained ~ 38%, 29% or zero activity when treated by 0.1, 0.5 or 1 µg/mL clavulanic acid. After another 24 hours culture, the clavulanic acid treated living non-IPN capsules producing Bla resumed the 87%, 53% and 53% activity. In contrast, living Bla sIPN capsules could partially stabilize the Bla function and maintained the ~ 66%, 45% or 11% of the enzymatic activity when treated by 0.1, 0.5 or 1 µg/mL clavulanic acid. All the clavulanic acid treated living Bla sIPN capsules resumed the activity after another 24 hours culture (**Supplementary Figure 9b**). Experiments were done in triplicate and data represented mean values ± s.d, with error bars = Standard Deviation (n=3). The x-axis is not to scale.
b. **Living non-IPN capsules producing Bla have a lower enzymatic activity compared with living Bla sIPN capsules under constant perturbation**. When clavulanic acid was supplemented for 24 hours (co-cultured with 0.1, 0.5 or 1 µg/mL clavulanic acid overnight, constant perturbation), living non-IPN capsules producing Bla maintained ~9%-45% of the enzymatic activity; in contrast, living Bla sIPN capsules maintained ~65%-86% of the enzymatic activity. Experiments were done in triplicate and data represented mean values ± s.d, with error bars = Standard Deviation (n=3).
c. **Capsules containing pure Bla did not recover the Bla activity after transient perturbation.** When clavulanic acid was supplemented for 10 mins (transient perturbation), capsules containing pure Bla remained ~ 24%, 9% or 2% of original activity when treated by 0.1, 0.5 or 1 µg/mL clavulanic acid. After another 24 hours culture, there is no recovery in the activity. The x-axis is not to scale. Experiments were done in triplicate and data represented mean values ± s.d, with error bars = Standard Deviation (n=3). The x-axis is not to scale.

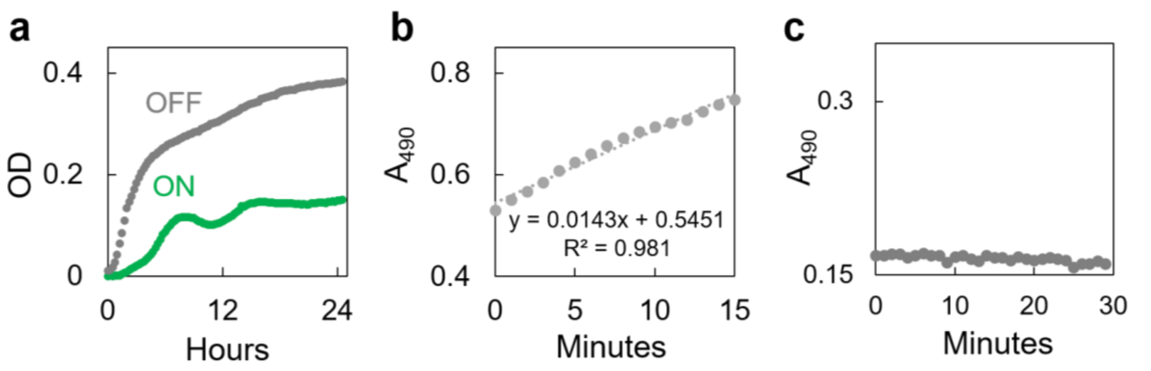

Supplementary Figure 11. The ePop function and expression of protein monomers are confirmed in Nissle strain.

a. **Nissle bacteria undergo controlled lysis when ePop circuit is on.** The circuit generated population-level lysis in Nissle bacteria cultured in M9 medium at 37°C, indicated by the green curve, compared with Nissle bacteria not carrying the circuit (gray curve).

b. **T_3-Bla expressed by Nissle exhibited Bla activity.** NI (T_3-Bla) cells were cultured in M9 medium for 24 hours. 5 μL of collected supernatant was diluted into 100 μL by PBS and then mixed by enzymatic reaction with the substrate nitrocefin (50 μM). The y-axis shows the absorbance at 490 nm. When the substrate is saturating, the initial slope of the absorbance is proportional to the activity of the Bla (see **Methods**).

c. **Nissle strain itself does not exhibit any detectable Bla activity.** 5 μL supernatant of Nissle strain overnight culture was diluted into 100 μL using PBS and mixed with nitrocefin to attain a final substrate concentration of 50 μM. The resulting absorbance at 490 nm was measured as a function of time. Our results show that in the absence of our circuit, Nissle strain itself also does not exhibited any detectable Bla activity by using the nitrocefin assay.

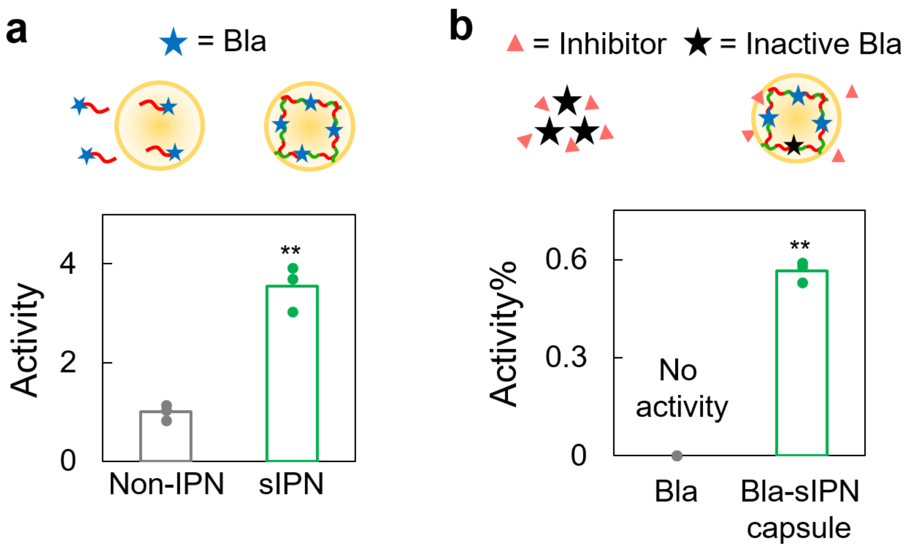

Supplementary Figure 12. Living Bla sIPN capsules stabilized anchored enzyme.

A. Living Bla sIPN capsules enhanced the activity of the anchored Bla *(Top: schematic; bottom: experimental data)*. NI (ePop/T_3-Bla) and NI (ePop/C_3) were mixed, encapsulated with chitosan and cultured in M9 for 24 hours. As a control, NI (ePop/C_3) was replaced with NI (ePop/T_3). The capsules were harvested and thoroughly washed by PBS before Bla activity assay. The data were normalized by the value of living non-IPN capsules producing Bla (gray bar = 1). Living Bla sIPN capsules fabricated by Nissle strain can anchor the Bla more efficient. Experiments were done in triplicate. The bar represents the mean value (n = 3). Significance is defined as $^{**}p < 0.01$ (p = 0.006) by the two-sided Student's two-sample t-test assuming unequal variances compared with the value for non-IPN.

B. Living Bla sIPN capsules protected Bla from inhibitors *(Top: schematic; bottom: experimental data)*. We added 0.5 µg/mL clavulanic acid, a Bla inhibitor, to both supernatant containing Bla and living Bla sIPN capsules and incubated for 10 mins before Bla activity assay. The data were normalized by the value of each sample without addition of Bla inhibitor. The activity of supernatant containing Bla dropped to zero (undetectable) after incubation with the inhibitor. Experiments were done in triplicate. The bar represents the mean value (n = 3). Significance is defined as $^{**}p < 0.01$ (p = 0.001) by the two-sided Student's two-sample t-test assuming unequal variances compared with the value for supernatant containing Bla.

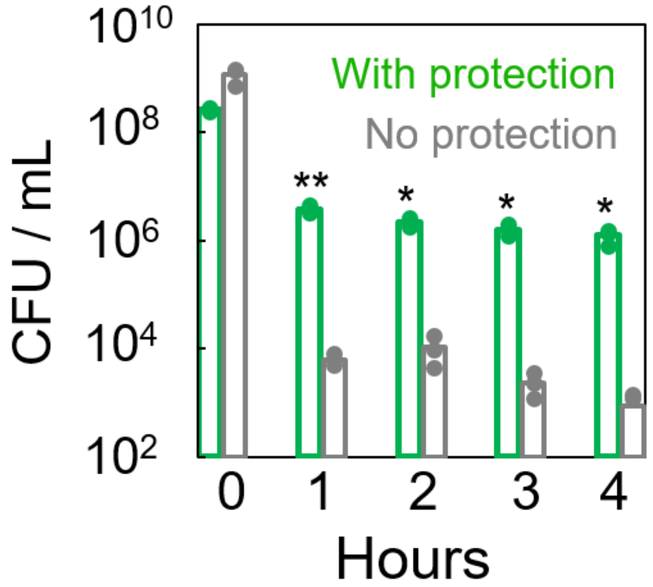

Supplementary Figure 13. Chitosan capsules protected cells from acidic environment. Unprotected or protected (cells encapsulated in capsules) NI (T_3-Bla) bacteria were incubated in the pH = 1 buffer for 0 (control), 1, 2, 3 or 4 hours, respectively. After incubation, the unprotected bacteria and cells in capsules were collected and plated for CFU counting. Experiments were done in triplicate. The bar represents the mean value (n = 3). Significance is defined as **$p < 0.01$ and *$p < 0.05$ ($p = 0.009$, 0.012, 0.02 and 0.03 for 1, 2, 3 and 4 hours) by the two-sided Student's two-sample t-test assuming unequal variances compared with the value for unprotected.

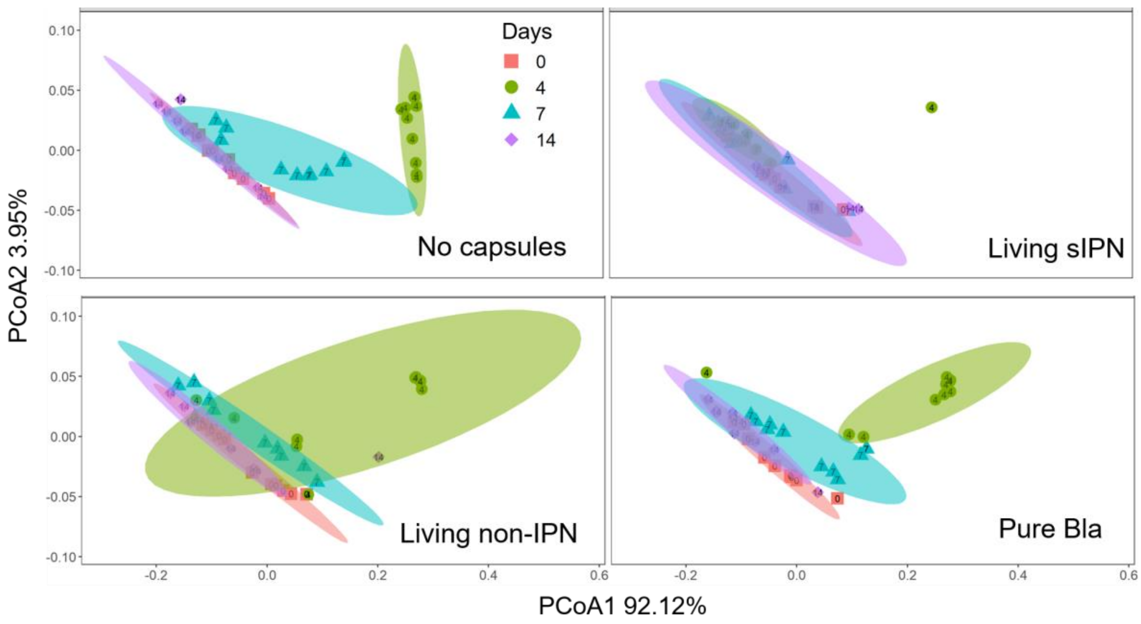

Supplementary Figure 14. Principal coordinate analysis (PCoA) of weighted UniFrac distances of the microbiota composition reveals that living Bla sIPN treatment was most effective in protection of the gut microbiome against ampicillin-mediated perturbation. Each panel represents a different group and each dot represents a mouse. The annotated ellipses (95% confidence intervals) fit to the clusters of 10 points. The colors indicate different time points. The red points represent the baseline microbiome. The living Bla sIPN group demonstrates the most significant protection on the composition of gut microbiota, especially on Day 4 and Day 7.

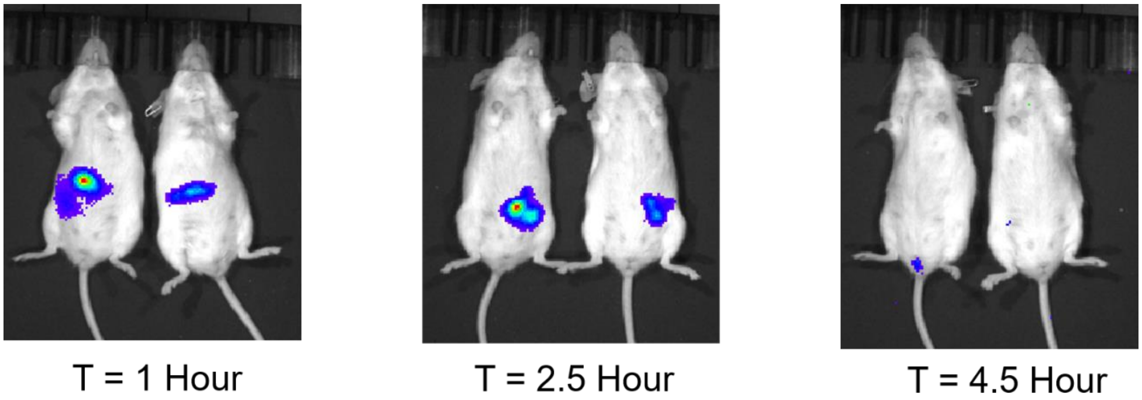

Supplementary Figure 15. Circulation dynamics of capsules inside mice. Cells (NI(luminescence)) were encapsulated by chitosan and cultured in M9 medium for 20 hours. The capsules were washed by PBS before administrated to the mice by gavage (~25,000 for each mouse). The mice were anaesthetized and imaged at different time points by IVIS Lumina III In Vivo Imaging System. The capsules stayed inside the gut for 4~5 hours before cleared out of the body.

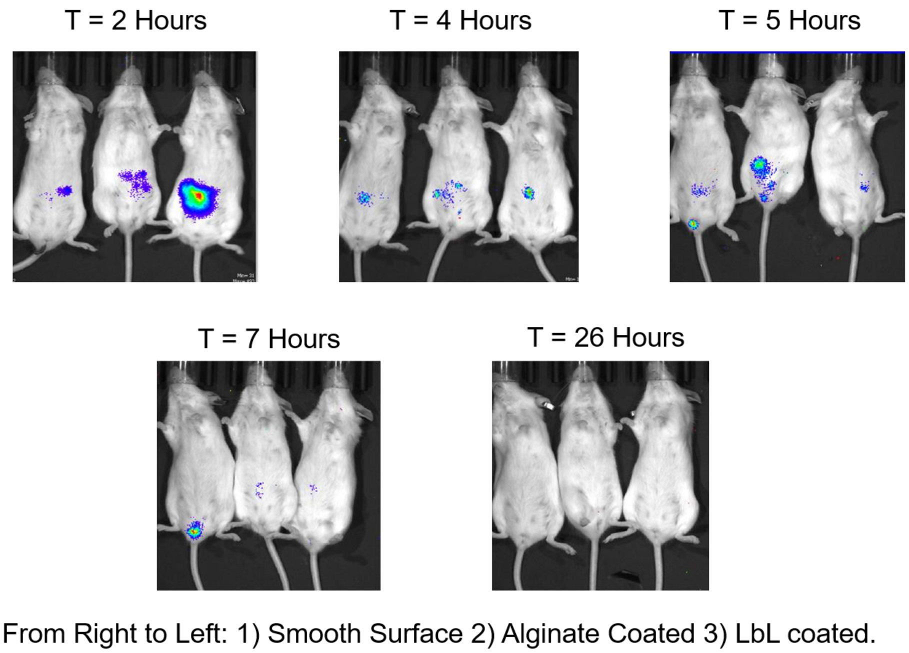

Supplementary Figure 16. Circulation dynamics of capsules was controllable by surface modification on the capsules. Cells (NI(luminescence)) were encapsulated by chitosan and cultured in M9 medium for 20 hours. The capsules were washed by PBS first, and further coated by alginate (alginate coated); or alginate and chitosan as a layer-by-layer modification (LbL capsules). The modification was implemented by first depositing negatively charged alginate, followed by positively charged chitosan with washing in between. ~25,000 capsules were administrated to each mouse. The mice were anaesthetized and imaged at different time points by IVIS Lumina III In Vivo Imaging System. From right to left, they are original capsules, alginate coated capsules and LbL coated capsules. Compared with chitosan capsules, the modified capsules (alginate coated and LbL coated) stay longer inside the gut (~6-7 hours) before cleared out of the body.

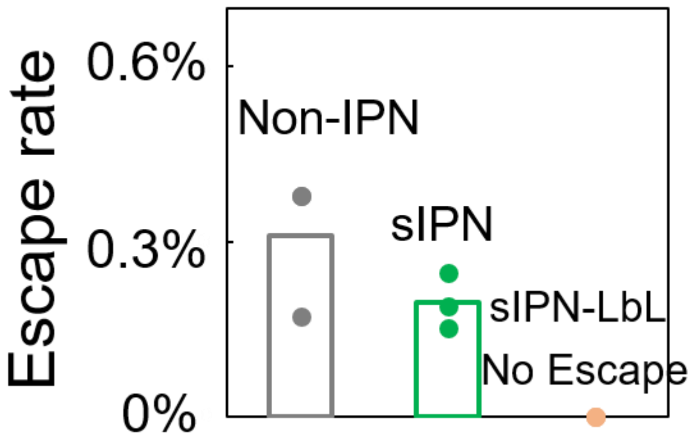

Supplementary Figure 17. Escape rate of bacteria from sIPN capsules was reduced by additional coating. Escape rate is defined as the percentage of the escape cells in terms of total cell number in capsules. It is calculated as dividing the number of the cells in the surrounding medium of living non-IPN or sIPN culture by the number of cells inside capsules (**Supplementary Figure 3c**). After LbL modification (see **Methods**), the escape rate of the cells decreases to zero for living sIPN capsules. The bar represents the mean value (n = 3). Experiments were done in triplicate.

Supplementary Tables

Supplementary Table 1: Bla activity (before normalization) generated by cell culture, living non-IPN capsules producing Bla and living Bla sIPN capsules. The fabrication of living non-IPN capsules producing Bla (MC(T_3-Bla) with MC(T_3)) and living Bla sIPN capsules (MC(T_3-Bla) with MC(C_3)) were described in methods. Both capsules were washed by PBS before enzymatic assay. For cell culture (supernatant), equal amount of cells (MC(T_3-Bla), same cell number with MC(T_3-Bla) with MC(C_3) or MC(T_3-Bla) with MC(T_3)) were inoculated into the same amount of nutrients and cultured for 24 hours. 5 μL of different samples were used to evaluate the Bla activity in the unit of OD_{490}/min (see **Methods**). The experiments were done in triplicate.

**Samples**	Supernatant	Living sIPN capsules	Living non-IPN capsules
Bla activity (OD_{490}/min)	0.012 ± 0.0010	0.022 ± 0.0012	0.007 ± 0.0015

Supplementary Table 2: Parametric P-values of the UniFrac distance calculated using the two-sided Student's two-sample t-test. We compared the results in **Figure 4a** (UniFrac) from pairs of different conditions of living Bla sIPN capsules and other three groups by using the two-sided Student's two-sample t-test (assuming unequal variances). Shortly after treatment (Day 4), the living Bla sIPN capsules provided statistically significant protection ($p<0.05$), compared with each of other three groups. The difference between different groups decreased over time: the microbiome composition was able to recover after the transient antibiotic treatment in each condition (with or without protection).

**P-values**	Day 4	Day 7	Day 14
No capsules	0.00001	0.03678	0.97188
Living non-IPN	0.01211	0.60405	0.91718
Pure Bla	0.00255	0.46766	0.99045

Supplementary Movies

Supplementary Movie 1: mCherry diffuses out in the control group with no IPN formation.
Supplementary Movie 2: Immobilization of mCherry due to the formation of sIPN.

Supplementary References

1 Marguet, P., Tanouchi, Y., Spitz, E., Smith, C. & You, L. Oscillations by Minimal Bacterial Suicide Circuits Reveal Hidden Facets of Host-Circuit Physiology. *Plos One* **5**, (2010).

2 Sun, F., Zhang, W. B., Mahdavi, A., Arnold, F. H. & Tirrell, D. A. Synthesis of bioactive protein hydrogels by genetically encoded SpyTag-SpyCatcher chemistry. *Proc Natl Acad Sci U S A* **111**, 11269-11274, (2014).

3 Andreu, N., Zelmer, A., Fletcher, T., Elkington, P. T., Ward, T. H., Ripoll, J., Parish, T., Bancroft, G. J., Schaible, U., Robertson, B. D. & Wiles, S. Optimisation of bioluminescent reporters for use with mycobacteria. *PLoS One* **5**, e10777, (2010).

4 Liu, Y., Zhang, K., Ma, J. & Vancso, G. J. Thermoresponsive Semi-IPN Hydrogel Microfibers from Continuous Fluidic Processing with High Elasticity and Fast Actuation. *ACS Appl Mater Interfaces* **9**, 901-908, (2017).
