# Supplementary material for: Living fabrication of functional semi-interpenetrating polymeric materials
Source: Nat Commun. 2021 Jun 8;12:3422. doi: 10.1038/s41467-021-23812-7 (PMC8187375; doi:10.1038/s41467-021-23812-7)
Supplement: Supplementary file 3 — Reporting Summary [file 41467_2021_23812_MOESM3_ESM.pdf]

nature research

Corresponding author(s): Lingchong You

Last updated by author(s): May 1, 2021

Reporting Summary

Nature Research wishes to improve the reproducibility of the work that we publish. This form provides structure for consistency and transparency in reporting. For further information on Nature Research policies, see Authors & Referees and the Editorial Policy Checklist .

Statistics

For all statistical analyses, confirm that the following items are present in the figure legend, table legend, main text, or Methods section.

n/a	Confirmed	
☐	☒	The exact sample size (n) for each experimental group/condition, given as a discrete number and unit of measurement
☐	☒	A statement on whether measurements were taken from distinct samples or whether the same sample was measured repeatedly
☐	☒	The statistical test(s) used AND whether they are one- or two-sided<br>*Only common tests should be described solely by name; describe more complex techniques in the Methods section.*
☒	☐	A description of all covariates tested
☒	☐	A description of any assumptions or corrections, such as tests of normality and adjustment for multiple comparisons
☐	☒	A full description of the statistical parameters including central tendency (e.g. means) or other basic estimates (e.g. regression coefficient) AND variation (e.g. standard deviation) or associated estimates of uncertainty (e.g. confidence intervals)
☐	☒	For null hypothesis testing, the test statistic (e.g. F, t, r) with confidence intervals, effect sizes, degrees of freedom and P value noted<br>*Give P values as exact values whenever suitable.*
☒	☐	For Bayesian analysis, information on the choice of priors and Markov chain Monte Carlo settings
☒	☐	For hierarchical and complex designs, identification of the appropriate level for tests and full reporting of outcomes
☒	☐	Estimates of effect sizes (e.g. Cohen's d, Pearson's r), indicating how they were calculated

Our web collection on statistics for biologists contains articles on many of the points above.

Software and code

Policy information about availability of computer code

Data collection	The data were collected using the commercial equipments and related softwares shown in Methods.
Data analysis	Alignment and demultiplexing of raw 16S ribosomal RNA sequencing data was performed with QIIME2 (version 2019.7). Images from microscopy were processed by ImageJ(1.52a/Java 1.8.0_112). Excel(2013) and R(3.6.1) were used for figure generation.

For manuscripts utilizing custom algorithms or software that are central to the research but not yet described in published literature, software must be made available to editors/reviewers. We strongly encourage code deposition in a community repository (e.g. GitHub). See the Nature Research guidelines for submitting code & software for further information.

Data

Policy information about availability of data

All manuscripts must include a data availability statement. This statement should provide the following information, where applicable:

- Accession codes, unique identifiers, or web links for publicly available datasets
- A list of figures that have associated raw data
- A description of any restrictions on data availability

The authors declare that the source data processed for figures generation in this study are available within the paper and its supplementary files. The data underlying Figures 3a-c, 4a-b, as well as Supplementary Figures 3a-c, 8a-c, 9b, 10a-c, 11a-c, 12a-b, 13, 14 and 17 are provided as source data file. Any additional information is available upon request.

Field-specific reporting

Please select the one below that is the best fit for your research. If you are not sure, read the appropriate sections before making your selection.

Life sciences study design

All studies must disclose on these points even when the disclosure is negative.

Sample size	For animal experiments, 10 mice were used as replicates in each group. The rest experiments (when replicates were shown) were performed with 3 biologically independent samples. The sample sizes were chosen based on the scale of the project and consistency with other similar published studies. No sample size calculation was performed. Our results suggested that the sample sizes we chose were sufficient to test the theoretical predictions.
Data exclusions	No data were excluded.
Replication	Each experiment was repeated at least three times with similar results, suggesting the robustness of our conclusions.
Randomization	40 six-week-old BALB/cJ male mice was randomly divided into 4 groups (10 mice in each group). For experiments that need overnight culture, clones were picked randomly.
Blinding	Investigators were not blinded. Knowledge of a samples identity did not affect the experimental conclusion.

Reporting for specific materials, systems and methods

We require information from authors about some types of materials, experimental systems and methods used in many studies. Here, indicate whether each material, system or method listed is relevant to your study. If you are not sure if a list item applies to your research, read the appropriate section before selecting a response.

Materials & experimental systems

n/a	Involved in the study	
☒	☐	Antibodies
☒	☐	Eukaryotic cell lines
☒	☐	Palaeontology
☐	☒	Animals and other organisms
☒	☐	Human research participants
☒	☐	Clinical data

Methods

n/a	Involved in the study	
☒	☐	ChIP-seq
☒	☐	Flow cytometry
☒	☐	MRI-based neuroimaging

Animals and other organisms

Policy information about studies involving animals; ARRIVE guidelines recommended for reporting animal research

Laboratory animals	We used 40 six-week-old BALB/cJ male mice. Mice were housed under standard conditions with a 12 h light–dark cycle at 20–26 °C (daily temperature difference < 4 °C), humidity 40 to 70%, and free access to food (GB 14924.3-2010 feed formula) and water.
Wild animals	No wild animals were used in the current study.
Field-collected samples	No field-collected samples were used in the current study.
Ethics oversight	All animal experiments were approved by the Institutional Animal Care and Use Committee at Shenzhen Institutes of Advanced Technology (permit number: SIAT-IACUC-191230-HCS-DL-A0979).

Note that full information on the approval of the study protocol must also be provided in the manuscript.
