# Supplementary material for: Living fabrication of functional semi-interpenetrating polymeric materials
Source: Nat Commun. 2021 Jun 8;12:3422. doi: 10.1038/s41467-021-23812-7 (PMC8187375; doi:10.1038/s41467-021-23812-7)
Supplement: Supplementary file 4 — Description of Additional Supplementary Files [file 41467_2021_23812_MOESM4_ESM.docx]

**Description of Additional Supplementary Files**

**Supplementary Movie 1:** mCherry diffuses out in the control group with no IPN formation.

**Supplementary Movie 2:** Immobilization of mCherry due to the formation of sIPN.
